# Comparing genotype and chemotype of *Fusarium graminearum* from cereals in Ontario, Canada

**DOI:** 10.1371/journal.pone.0216735

**Published:** 2019-05-09

**Authors:** Trinda Crippin, Justin B. Renaud, Mark W. Sumarah, J. David Miller

**Affiliations:** 1 Department of Chemistry, Carleton University, Ottawa, Ontario, Canada; 2 London Research and Development Center, Agriculture and Agri-Food Canada, London, Canada; Universita degli Studi di Pisa, ITALY

## Abstract

*Fusarium graminearum* is responsible for production of the mycotoxin deoxynivalenol (DON) on maize and wheat in Ontario, Canada. It has been understood since the early 1980s that in most parts of Canada, the predominant chemotype of *F*. *graminearum* is 15ADON, and not the 3ADON chemotype mainly found in Europe and Asia. The discovery of *F*. *graminearum* strains that did not produce DON but the structurally related 7-α hydroxy, 15-deacetylcalonectrin (3ANX) and its hydrolysis product 7-α hydroxy, 3,15-dideacetylcalonectrin to (NX) demonstrated that we still have a lot to learn about this well studied but complicated fungus. We conducted a survey of maize and wheat samples from Ontario farms. In the 2015 crop year, we isolated 86 strains and tested a representative subset of 20 using the published genetic probes for assessing genotype. We also developed a targeted LC-MS/MS method for the identification and quantitation of known toxins from this species to determine chemotype. The results showed that 80% of our strains produced some 3ANX in addition to 15ADON and one strain produced 3ANX and no 15ADON. Comparison of chemical data with genotyping revealed that in more than 50% of the cases there was no clear agreement. These data demonstrate the importance of chemical analysis for understanding the toxigenic potential of strains, especially using a LC-MS method that is capable of differentiating 3ADON and 15ADON. For this collection, genotyping of isolates did not produce reliable information on the chemotype. This is the first report of 3ANX toxin production concurrently with 15ADON and suggests that the 3ANX producers in North America likely originated from the 15ADON background.

## Introduction

*Fusarium graminearum* and related species that produce the mycotoxin deoxynivalenol (DON) are important pathogens of maize and small grains in temperate areas worldwide [1,2]. The diseases Fusarium head blight (FHB) and Gibberella ear rot (GER) have been observed for hundreds of years. Further, it was reliably known that grain affected by these diseases contained extractable toxins since the 1930s. However, the structure of deoxynivalenol (DON) was not resolved until 1973 by the Japanese scientist Takumi Yoshizawa using strains of *F*. *graminearum* isolated from grain that had resulted in human toxicosis [3]. The Japanese strains produced DON via 3, 15-diacetyl-DON to the monoacetate 3-acetyl-DON (3ADON) [4,5].

**Fig 1:**
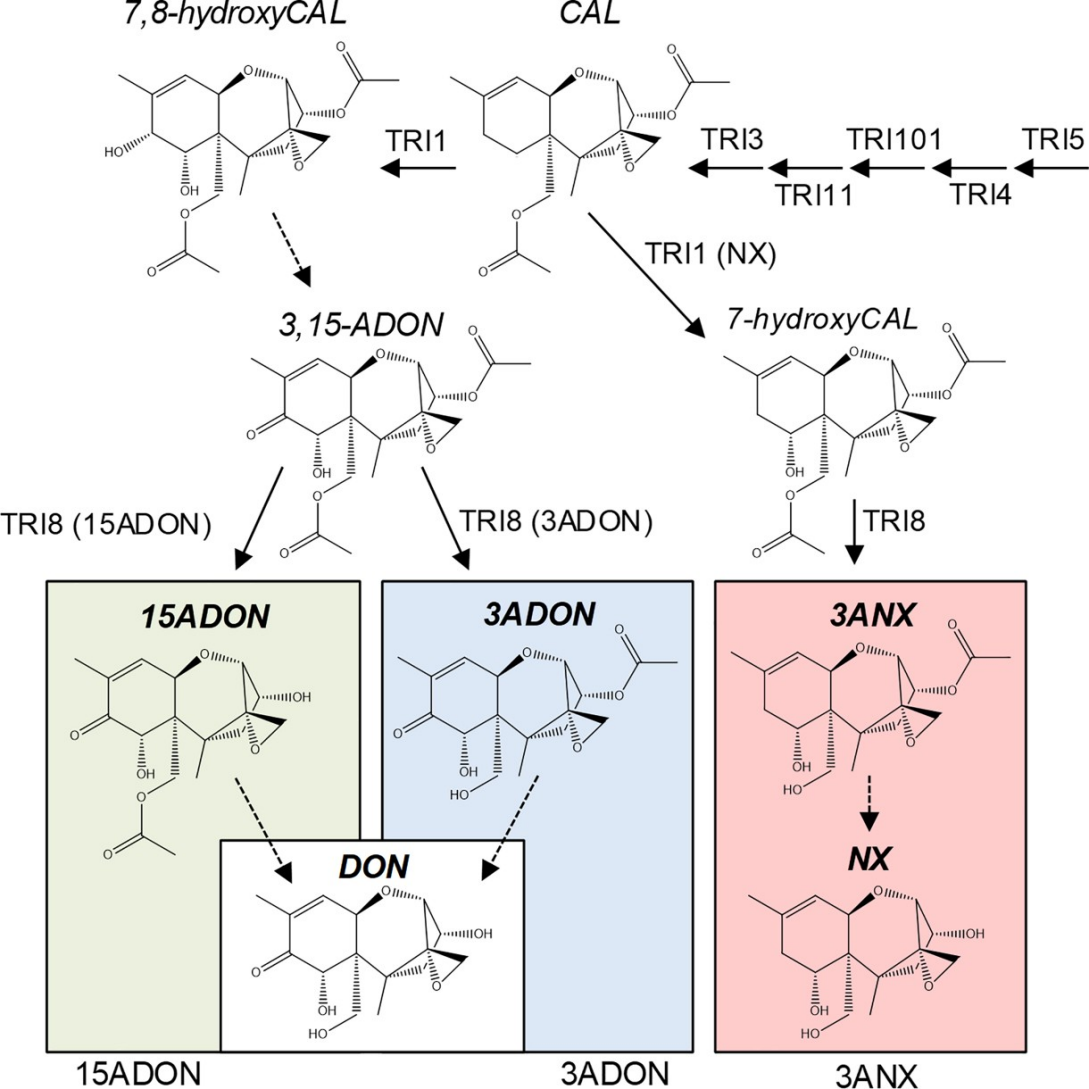
Putative biosynthetic pathways beginning from calonectrin (CAL) leading to different *F*. *graminearum* trichothecenes discussed in this study. 7,8-dihydroxycalonectrin (7,8-hydroxyCAL); 7-hydroxycalonectrin (7-hydroxyCAL); 3,15-diacetyldeoxynivalenol (3,15-ADON); 15-acetyldeoxynivalenol (15ADON); 3-acetyldeoxynivalenol (3ADON); deoxynivalenol (DON); 3ANX (NX-2 toxin); NX (NX-3 toxin).

In response to the catastrophic epidemics of FHB in Ontario from 1980, research began on the toxins produced by Canadian strains of *F*. *graminearum*. This revealed that all strains from southern [6] and eastern Ontario [7] examined at that time produced DON predominately *via* a 15-acetyl-DON (15ADON) [8] precursor rather than 3ADON as with the Japanese strains (Fig 1). This is caused by altered deacetylase activities [9], resulting from sequence variations in the TRI8 enzyme [10]. This discovery led to work on identifying the ‘chemotype’ of *F*. *graminearum* strains worldwide by determining the major monoacetate precursor produced by different populations [11]. Early analysis of culture extracts from strains collected on maize in Minnesota revealed that the vast majority produced 15ADON and about 20% were reported to be 3ADON [12,13]. Chemical analysis of culture extracts from strains collected in China, Europe and Mexico in the mid-1980s revealed that there were strong geographic patterns with respect to chemotype. Of thirteen *F*. *graminearum* strains isolated from wheat across nine provinces in China, 11 were 3ADON producers with two strains producing 15ADON [11]. Analysis of a larger collection from the same provinces (47 strains) found a similar pattern, with 3ADON producers most often isolated from warmer regions. Those isolated from wheat in the somewhat cooler provinces (Henan, Shaanxi, Heilongjiang and mountain area of Fujian) were 15ADON producers [14]. Mirocha et al. [13] reported that strains from China, Australia and New Zealand were mainly 3ADON producers, whereas strains from sites in Mexico (Toluca and Jalisco States) were 15ADON producers [11]. In the 1980s, *F*. *culmorum* was more common than *F*. *graminearum* in cooler wheat growing areas such as the UK and most of continental Europe [15,16]. These strains produced 3ADON [11,17–20]. However, strains of *F*. *graminearum* from Portugal and the former Yugoslavia were reported as 15ADON producers [18].

With increased understanding of the specific biosynthetic differences between *F*. *graminearum* chemotypes (Fig 1), PCR-based systems were developed to differentiate the corresponding genotypes [21]. Initially, PCR based screening of the toxigenic potential of all or a high percentage of strains from cultures or infected wheat were strengthened by chemical analysis [22–24]. As time passed, it became less common to report both PCR and chemical analysis for each strain. For example, studies on *F*. *graminearum* populations in Canada [25], France [26], Norway [27], the UK [28] and Uruguay [29,30] reported no chemical data; the chemotypes were inferred from the genotypes. Other studies performed chemotype analysis on a modest percentage of strains examined e.g. from Brazil (12%) [31], Japan (3%) [32], Ontario (19%) [33] and the USA (10%) [34].

A genetic-only approach to infer chemotype and thus the toxigenic potential of filamentous fungi suffers from two key limitations. Firstly, it has been long known that in contrast to the enzymes of primary metabolism in filamentous fungi, which are exquisitely specific, those of secondary metabolism are typically less specific [35]. This means that strain to strain variations in the accumulation of precursor secondary metabolites can result in a variety of end products along what came to be described as a metabolic grid [36–38]. Studies have shown that these differences in substrate specificity of biosynthetic enzymes means that several pathways exist even for the same compound [38–40]. Secondly, as with sequence differences in the *TRI8* gene results in populations that produce either 3ADON or 15ADON, genetic variations that arise within other genes involved in secondary metabolism can lead to the production of different chemical end-products.

About a decade ago, Gale and co-workers discovered a population of *F*. *graminearum* in the Midwest United States that was genotyped as 3ADON (*TRI3*, *TRI12* assay) but did not produce 3ADON, 15ADON, DON, fusarenone X (4ANIV), nor nivalenol (NIV) [41]. Closer investigation of these strains determined that this population produced a new trichothecene that was identical to 3ADON but lacked the C-8 keto oxygen (Fig 1) [42]. This 3-acetyl NX toxin (3ANX), also described as NX-2 toxin, is believed to result from a sequence variation in the *TRI1* gene. The TRI1 enzyme in *F*. *graminearum* is a P450 oxygenase responsible for the addition of hydroxyl groups to the C-7 and C-8 positions of calonectrin [43]. The analogue of TRI1 found in *F*. *sporotrichioides* adds a single hydroxyl group to the C-8 position [44] and it is reasonable that the TRI1 in the NX population adds a single hydroxyl group to the C-7 position (Fig 1) [42]. After this discovery, most studies only provided chemical analysis of strains that genetic tests inferred were NX chemotypes or strains where the genetic tests were ambiguous [45,46]. Other labs likely did not have reliable standards of 3ANX and NX, which are not yet commercially available. Some studies report 3ADON and 15ADON as the sum of the two rather than individually [47,48], even though analytical methods to differentiate the acetates have been reported [49]. In such studies, the failure to compliment genetic tests with chemotyping data led to assumptions about the toxins produced. For clarity, we have chosen to maintain the NX naming designation for these compounds as described by Varga et al. [42] but to use the common names 3ANX (NX-2) and NX (NX-3) analogous to 3 or 15ADON and DON.

Farmers in Ontario produce 50% of maize and most of the soft wheat produced in Canada. Spanning 1000 km, growing conditions vary from 3500 to 2900 corn heat units with average morning June RH from 91 to 81%, respectively in a wheat, maize and soy rotation. Each year, grain samples are taken throughout the cereal producing areas of the Province (~200,000 km^2^). Working from ~500 samples of the grain from 94 farms, kernels were plated on a semi-selective medium for *Fusarium* resulting in a large number of isolates of *F*. *graminearum*. A proportionate subsample of the strains collected were subjected to comprehensive chemical analysis specifically designed to distinguish all significant *F*. *graminearum* metabolites and the available probes used for genotyping the strains The purpose of this report is to discuss the analytical methods used and the relationship between the genotype and chemotype.

## Materials and methods

### Isolates

In 2015, a total of 141 shelled maize samples were collected from 28 farms and 398 wheat samples from 66 farms. The GPS coordinates of each sample were recorded. The samples were collected by the Ontario Corn Committee, Kent Corn Committee, experimental plots at the University of Guelph Ridgetown Campus as well as samples collected from annual ear rot and Fusarium Head Blight/ Gibberella ear rot surveys conducted by the Ontario Ministry of Food and Agriculture, and the University of Guelph, Ridgetown Campus.

Three *F*. *graminearum* positive control strains were obtained from Agriculture and Agri-Food Canada as positive controls. The 15ADON positive control strain, *F*. *graminearum* DAOMC 180378, was isolated from maize in Ottawa, Ontario in 1979 [7,8]. The 3ADON strain, *F*. *graminearum* DAOMC 242075, was isolated in 2011 from red spring wheat in New Brunswick. The 3ANX strain, *F*. *graminearum* DAOMC 242077, was isolated from soft red winter wheat in Annapolis, Nova Scotia, also in 2011.

### Isolation and identification

*F*. *graminearum* strains were isolated from grains in the bagged samples of wheat and maize (90-100g). The kernels were surface sterilized and plated onto PCNB medium (peptone; HI-Media Laboratories, 15 g; K_2_HPO_4_ 1 g; MgSO_4_•7H_2_O 15 g; pentachloronitrobenzene 0.75 g; streptomycin 0.3 g; neomycin 0.12 g per L ultrapure water, Barnstead Nanopure Diamond). From the well-mixed grains in each sample, 10 kernels of maize or 14 kernels of wheat were plated. The plates were incubated at 26°C for 7 days in darkness. All fungal colonies that appeared to be *Fusarium* were then transferred to Potato Dextrose Agar (Hardy Diagnostics, #C6622), and incubated at 26°C in darkness. After one week, each *F*. *graminearum* isolate was identified based on morphological characteristics and stored at 4°C. From the 2015 season, 77 strains were isolated from samples of wheat from 25 farms and 9 from maize samples from two farms.

Aerial mycelium from a fresh plate of each isolate was used to extract genomic DNA with the Macharey-Nagel Nucleospin 96 Plant-II kit (GmbH & Co. KG #740663.4). All PCR reactions were amplified in a T100 Thermal Cycler (Bio-Rad Technologies, Inc., CA, USA). 10 μL of amplified PCR products were run in 1.5% (*wt/vol*) agarose gels, pre-stained with ethidium bromide to confirm amplification. Gel photographs were taken on MultiImage Light Cabinet (Alpha Innotech, Thermo Fisher Scientific, CA, USA). Invitrogen PCR SuperMix (Thermo Fisher Scientific, CA, USA) was used for all PCR reactions. PCR primers were synthesized by Integrated DNA Technologies (Integrated DNA Technologies, Coralville, IA, Canada).

From this collection, 20 strains were selected to represent each location where *F*. *graminearum* was recovered representing an area of 20,000 km^2^ (S1 Table). These samples represent a large collection area (~20,000 km^2^) representing differing weather patterns. The attempt was to represent as large a geographic area as possible. The subset included 18 strains isolated from wheat and two from maize. These cultures were deposited in the Canadian Collection of Fungal Cultures (DAOMC 251903–251922) with GenBank (accession numbers MH108123-MH108142). To identify the phylogenetic clade of *F*. *graminearum*, a portion of the translation elongation factor (TEF) gene was amplified by PCR primer sets EF-1 and EF-2 [50,51] and sequenced by Génome Québec Innovation Centre (Montreal, Québec). Sequencing of the TEF gene was performed using Sanger sequencing in both forward and reverse directions. The raw sequences were aligned and the identities were confirmed using BLAST (blast.ncbi.nlm.nih.gov/Blast.cgi).

A multiplex PCR assay targeting *TRI3* and *TRI12* was used to differentiate between 15ADON and 3ADON producers of *F*. *graminearum* according to Ward et al. [23] following the protocol described in Starkey et al. [22]. The *TRI8* PCR assay developed by Alexander et al. was also tested [10]. In order to differentiate between 3ADON and 3ANX producers, we tested the PCR-RFLP assay developed by Liang et al. [45] and a *TRI1* allele specific amplification (Toomajian, personal communication; [52]). The *TRI1* allele specific PCR assays used a common reverse primer (*TRI1-*R; 5’-TTCCTGCAGGGGCTTGATG-3’) and differing forward primers for the 3ADON (5’-AATGCTCGCGAACTAATCAC-3’), and 3ANX (5’AATGCTAGCGAAATGATCAA-3’) genotypes. Each 29 μL PCR reaction consisted of 22.5 μL of Invitrogen PCR SuperMix (Thermo Fisher Scientific), 2 μL of 10 μM for each forward and reverse primer, and 2.5 μL of fungal genomic DNA. The thermal cycling conditions were 94°C for 2 min, 40 cycles of 94°C for 30s, 49.5°C for 30s, 68°C for 1 min, and a final extension at 68°C for 5 min. A GeneRuler100-bp DNA size ladder (Thermo Fisher Scientific) was used to score the molecular size of each amplicon.

### Fermentation and extraction

The toxigenic potential of each of the 20 strains was determined by analysis of extracts obtained from agar plate cultures after Visagie et al. [53]. Briefly, all strains were grown on 9 cm polystyrene Petri dishes on both 2% malt extract agar (MEA) and Glucose Yeast Extract Peptone (GYEP) agar at 28°C for 7 d. Three agar plugs were removed with a sterilized 7 mm cork borer from each of the three colonies on the plate for a total of 9 plugs. The plugs were transferred to a 13 mL polypropylene tube. Ethyl acetate (2 mL) was added to the tubes and vortexed for 30 s, followed by 15 minutes of sonication at 25°C, followed by vortexing for 30 s. The supernatants were transferred to clean amber vials and dried under a gentle stream of nitrogen. Extracts were reconstituted in 1 mL of 8:2 methanol:water and filtered into 2 mL amber glass HPLC vials using a 0.45 μm PVDF syringe filter.

Liquid fermentation culture flasks were also tested; the first stage medium comprised molasses 20 g, glucose 30 g, fish-meal 15 g, Pharmamedia 15 g per L of ultrapure water; 50 mL in 250 Erlenmeyer flasks. A MEA plug from a plate of each strain was macerated in sterile distilled water (27 mL). An aliquot (2.5 mL) was used to inoculate each of two flasks with test strains. The cultures were placed on a rotary shaker (3.81 cm throw) at 220 rpm at 28°C for 72 h in darkness. The resulting mycelium from the starter culture was macerated and 2.5 mL aliquots were added to six 250 mL Erlenmeyer flasks containing 50 mL of GYEP medium (glucose 10 g, yeast extract, VWR, 1 g; peptone 1 g per L ultrapure water; pH 6.5). These cultures were incubated at 28°C for 10 days in darkness [8,54]. The filtrates were loaded on a Chem Elut column (Agilent 100 mL; #12198010) and extracted with three 100 mL portions of ethyl acetate. The resulting extracts were reduced to dryness and stored at -20°C until analysis.

### LC-MS/MS chemical analysis

Chemical standards of NIV, 4ANIV, 3ADON, 15ADON were purchased from Romer Labs (BC, Canada). 3ANX was isolated from cultures of *F*. *graminearum* DAOMC 242077 and a portion was converted to NX using potassium hydroxide [55].

All MS data were collected with a Q-Exactive Quadrupole Orbitrap mass spectrometer (Thermo Scientific, MA, USA) coupled to an Agilent 1290 high-performance liquid chromatography system. Mycotoxins were resolved using a Zorbax Eclipse Plus RRHD C18 column (2.1× 100 mm, 1.8 μm; Agilent Technologies, CA, USA) maintained at 35°C. The mobile phase was comprised of water with 0.1% formic acid (A), and acetonitrile with 0.1% formic acid (B) (Optima grade, Fisher Scientific, NJ, USA). The gradient began by holding mobile phase B at 5% for 30 seconds, before increasing to 22% over 30 seconds. In order to resolve 3ADON and 15ADON, mobile phase B was then increased to 37% over 3 minutes before increasing to 100% over 1.5 minutes and held for two minutes before returning to 5% over 45 seconds. Five μL injections were used with a flow rate of 0.3 mL/min. Compounds were analyzed by a combination of full MS, and MS/MS at a resolution of 17, 500, automatic gain control (AGC) of 1 ×10^6^ maximum injection time (IT) of 64 ms and isolation width of 1.2 amu. Full MS scans between mass range *m/z* 100–1000, were acquired concurrently throughout the entirety of the LC run to allow for retrospective analysis and putative detection of other fungal metabolites. The full MS scan settings were AGC target 3 ×10^6^, max IT of 64 ms and resolution of 17, 500. The APCI settings were: spray current, 3.5 kV; capillary temperature, 300°C; sheath gas, 32.00 units; auxiliary gas, 10.00 units; probe heater temperature, 250°C; S-Lens RF level, 50.00.

## Results

### *F*. *graminearum* on maize and wheat samples in the 2015 crop year

A total of 141 samples of shelled maize were collected from 28 farms from the 2015 crop year. *F*. *graminearum* was detected in 6% of maize kernel samples and 7% of maize producing farms. A total of 398 grain samples were collected from 66 wheat-producing farms in 2015. *F*. *graminearum* was detected in 19% of grain samples overall and 39% of wheat producing farms, all from counties in southern Ontario (see above). These strains were confirmed as *F*. *graminearum* by the TEF gene after O’Donnell et al. [50]. In this crop year, all the strains of *F*. *graminearum* that were recovered came from the most southern counties of Ontario between Wellington and Kent Counties (43.9200° N, 80.0° W to 42.4° N, 82.1° W).

### Chemical analysis

The 20 representative isolates of *F*. *graminearum* were grown under three conditions (GYEP agar, MEA agar and liquid culture) and screened using our trichothecene specific LC-MS/MS method. For the liquid fermentation cultures, the concentration of each analyte was also quantitated (Table 1). The 15ADON, 3ADON and 3ANX control strains produced their predicted monoacetate trichothecenes in all conditions. In all three tested media, 15% (n = 3) of the 20 representative isolates of *F*. *graminearum* produced 15ADON and no 3ANX, while only 5% (n = 1) produced 3ANX and no 15ADON. Interestingly, we found that 80% (n = 16) of the isolates examined, co-produced detectable levels of both 15ADON and 3ANX in at least one medium. Some same-strain differences were observed in different growth media. Under the growth conditions where the 16 strains co-produced 15ADON and 3ANX, the amount of 15ADON was greater than the amount of 3ANX in 88%, 100% and 63% for MEA agar, GYEP agar and liquid culture respectively.

**Table 1:** Genotype and chemotype of the strains tested.

DAOM	Consensus genotype ^1^	Chemical analysis^2^	Genetic Assays					Chemical Assay				
			Starkey^3^	Alexander^4^	Liang^5^	Toomajian^6^		analytes detected			ug/L filtrate	
			*TRI3/TRI12*	*TRI8*	*TRI1*	ADON	ANX	GYEP	MEA	liquid	15ADON	3ANX
180378*	**15ADON**	**15ADON**	15ADON	15ADON	ADON	+	+	15ADON	15ADON	15ADON	+++	-
242075*	**3ADON**	**3ADON**	3ADON	3ADON	ADON	+	-	3ADON	3ADON	3ADON	+	-
242077*	**3ANX**	**3ANX**	3ADON	3ADON	ANX	-	+	3ANX	3ANX	3ANX	-	+
*251904*	15ADON	15ADON	15ADON	15ADON	ADON	+	+	15ADON	15ADON	15ADON	534	nd
*251910*	15ADON	15ADON	15ADON	15ADON	ADON	+	+	15ADON	nd	15ADON	44	nd
*251917*	**-**	15ADON	15ADON	15ADON	-	+	+	15ADON	15ADON	15ADON	110	nd
*251908*	15ADON	15ADON,3ANX	15ADON	15ADON	ADON	+	+	15ADON, 3ANX	nd	15ADON, 3ANX	63270	401
*251906*	15ADON	15ADON,3ANX	15ADON	15ADON	ADON	+	+	15ADON, 3ANX	15ADON, 3ANX	15ADON, 3ANX	2990	49
*251907*	15ADON	15ADON,3ANX	15ADON	15ADON	ADON	+	+	15ADON, 3ANX	nd	15ADON, 3ANX	207	207
*251921*	15ADON	15ADON,3ANX	15ADON	15ADON	ADON	+	+	15ADON	15ADON	15ADON, 3ANX	123	119
*251913*	15ADON	15ADON,3ANX	15ADON	15ADON	ADON	+	+	15ADON	15ADON	15ADON, 3ANX	77	39
*251916*	15ADON	15ADON,3ANX	15ADON	15ADON	ADON	+	+	15ADON	15ADON, 3ANX	15ADON, 3ANX	51	1707
*251918*	**-**	15ADON,3ANX	15ADON	15ADON	-	-	+	15ADON, 3ANX	15ADON, 3ANX	15ADON, 3ANX	46120	137
*251909*	**-**	15ADON,3ANX	15ADON	15ADON	ADON	-	+	15ADON	nd	15ADON, 3ANX	994	31
*251914*	**-**	15ADON,3ANX	15ADON	-	ADON	+	+	15ADON	3ANX	15ADON, 3ANX	875	56
*251912*	**-**	15ADON,3ANX	15ADON	-	ADON	+	-	15ADON	15ADON	15ADON, 3ANX	271	1174
*251903*	**-**	15ADON,3ANX	15ADON	15ADON	ADON	+	-	15ADON, 3ANX	15ADON	15ADON, 3ANX	66	44
*251920*	**-**	15ADON,3ANX	15ADON	-	ADON	+	+	15ADON, 3ANX	15ADON, 3ANX	15ADON	50	nd
*251915*	**-**	15ADON,3ANX	15ADON	-	ADON	+	+	15ADON, 3ANX	15ADON	15ADON, 3ANX	34	29
*251922*	**-**	15ADON,3ANX	15ADON	15ADON	ADON	-	-	15ADON	15ADON, 3ANX	15ADON, 3ANX	31	451
*251919*	**-**	15ADON,3ANX	15ADON	-	ADON	+	-	15ADON	15ADON	15ADON, 3ANX	17	156
*251905*	**-**	15ADON,3ANX	15ADON	15ADON	ADON	+	-	15ADON, 3ANX	nd	3ANX	nd	3831
*251911*	15ADON	3ANX	15ADON	15ADON	ADON	+	+	nd	nd	3ANX	nd	380

* genotype/chemotype strain

^1^ Defined as matching all genetic assays of the defined strains.

^2^ Spectrum of target analytes detected in three growth conditions.

^3^ Fungal Genet Biol 44(11):119

^4^ Fungal Genet Biol. 48(5):485

^5^ Fungal Genet Biol. 73:83

^6^ see text

**Fig 2:**
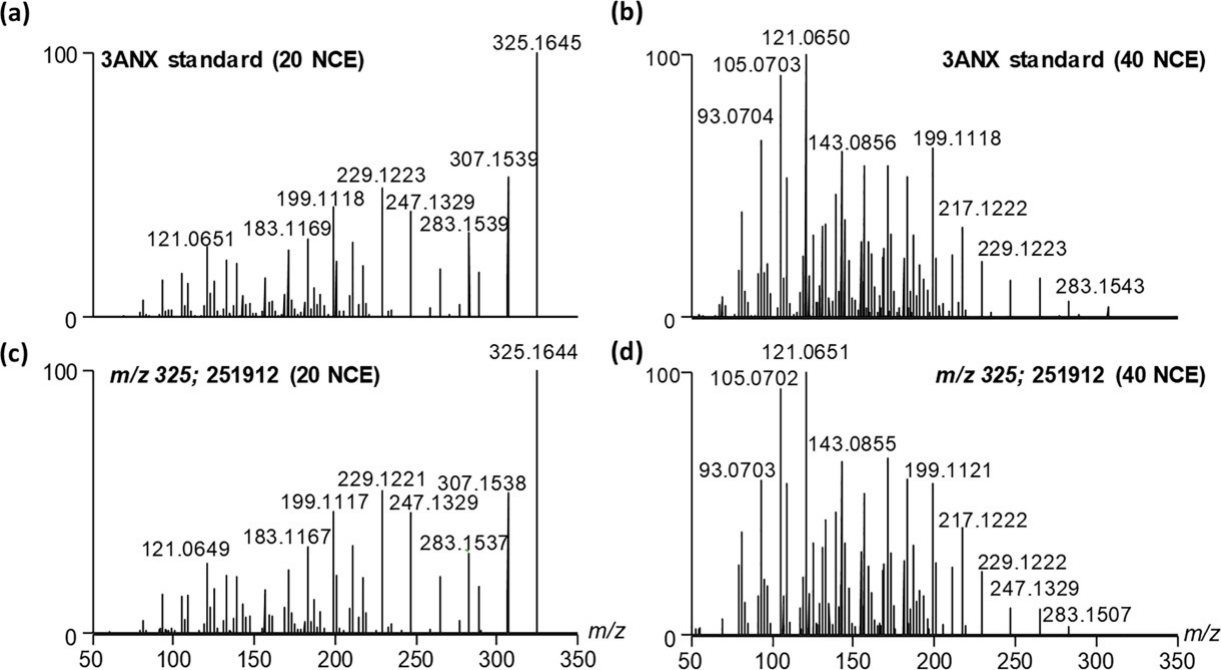
Comparison of the MS/MS spectra of a 3ANX standard at (a) 20 NCE and (b) 40 NCE with the putative 3ANX (c,d) detected culture filtrates of strain DAOMC 251912.

**Fig 3:**
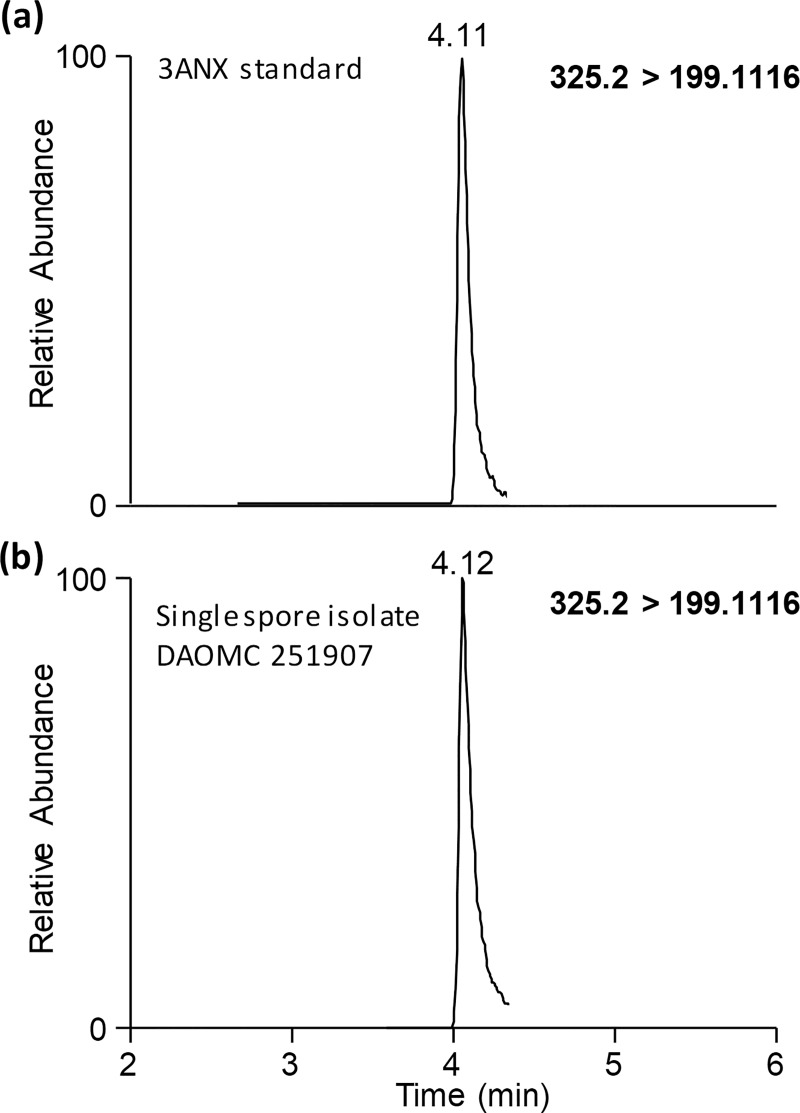
LC-MS/MS extracted ion chromatogram of (a) 3ANX standard with the (b) 3ANX detected in culture filtrates of DAOMC 251907.

To confirm that 3ANX was present in the same fermentation cultures as 15ADON, the MS/MS spectra of a 3ANX standard was compared to 3ANX detected in strain DAOMC 251912 at two different collision energies (Fig 2). Not only are the accurate masses of the product ions observed in MS/MS spectra from the standard and the compound in the extracts identical (< 3 ppm) their relative intensity ratios matched at both collision energies. To confirm that the co-detection of both 15ADON and 3ANX in the majority of strains was not the result of mixed cultures, single spore isolates of DAOMC 251907 were produced and confirmed the co-production of both compounds (Fig 3). The majority of strains also produced zearalenone and sambucinol on one or more of the growth conditions tested.

### Concordance with probe results

The results of the genotype analysis are shown in Table 1. We found that all probes tested were consonant with the expected results for the positive control strains used in this study. The probes for *TRI3* and *TRI12* reported by Ward et al. [23] and Starkey et al. [22] as well as the *TRI8* assay reported by Alexander et al. [10] indicated that the well-studied 15ADON producing strain DAOMC 180378 represented the 15ADON genotype correctly. The 3ADON producing strain DAOMC 242075 also tested correctly as a 3ADON genotype for *TRI3*, *TRI12*, and *TRI8* assays. As expected, the 3ANX control strain DAOMC 242077 tested as a 3ADON genotype for *TRI3*, *TRI12*, and *TRI8* assays. The RFLP-PCR assay targeting *TRI1* [45] and a separate *TRI1* assay developed by Toomajian [52] are purported to discriminate between 3ANX and ADON chemotypes (15ADON or 3ADON). Both the RFLP-PCR and Toomajian assays resolved DAOMC 242077 as a 3ANX genotype. When applied to DAOMC 180378 and DAOMC 242075 positive control strains, the RFLP-PCR assay indicated a ADON genotype. The Toomajian *TRI1* assay correctly distinguished the 3ADON and 3ANX strains but suggested the 15ADON positive control strain was both an ADON and 3ANX genotype.

In contrast to the agreement observed in the positive control strains, we found that genotypes based on the literature probes from the systematic collection in Ontario generally did not align with all genetic assays for the positive control strains (Table 1). For 55% (n = 11) of strains, the combined probe results provided no absolute genotype consensus with any of the positive control strains. The 3ANX producer, DAOMC 251911, indicated a 15ADON genotype for the *TRI3*, *TRI12* and *TRI8* assays. The RFLP-PCR assay targeting *TRI1* resulted in ADON genotype, whereas the assay developed by Toomajian suggested that DAOMC 252911 was both a ADON and 3ANX genotype, which is consistent with the 15ADON control strain (DAOMC 180378). The three strains producing only 15ADON were DAOMC 251904, 251910 and 251917. The genetic analysis performed in this study obtained a consensus match with the 15ADON positive control strain for DAOMC 251904, 251910 however the RFLP-PCR assay could not identify DAOMC 251917 as an ADON nor a 3ANX genotype. Overall, there was agreement between the genotype and chemotype approximately half the time.

Strains that could produce both 15ADON and 3ANX in liquid culture were isolated from both wheat (n = 14) and maize (n = 2) including DAOMC 251906, 251907, 251908, 251913, 251916 and 251921. All strains resulted in 15ADON amplification for *TRI3* and *TRI12* assay. Although these six strains produced both 15ADON and 3ANX in liquid culture, the PCR results matched those obtained from the 15ADON strain (DAOMC 180378).

For the remaining 10 strains that produced both 15ADON and 3ANX in liquid culture, there was no evident genotype consensus against positive control strains (Table 1). Tests of DAOMC 251903 and 251905 resulted in a ADON genotype for *TRI1* RFLP-PCR assay and ADON genotype for the Toomajian assay. The *TRI3*, *TRI12* and *TRI8* assays indicated a15ADON genotype for DAOMC 251909. This strain resulted in ADON genotype for the RFLP-PCR assay, however indicated that it was the 3ANX genotype by the Toomajian *TRI1* PCR assay. Tests of strains DAOMC 251912 and 251919 resulted in ADON genotype for *TRI1* RFLP-PCR and the Toomajian assays, but did not amplify in the *TRI8* assay. DAOMC 251914, 251915 and 251920 did not amplify in the *TRI8* assay, but the RFLP-PCR assay and the Toomajian assays suggested the strain was the ADON genotype. The tests of DAOMC 251918 indicated the 15ADON genotype for the *TRI8* assay however did not amplify in the RFLP-PCR assay and resulted in 3ANX genotype for the Toomajian assay. For DAOMC 251922, the RFLP-PCR assay resulted in a ADON chemotype and the *TRI8* assay indicated 15ADON (Table 1), however no amplification was found using the Toomajian assay for either the ADON or 3ANX genotype.

## Discussion

A decade ago, Dr. Anne Desjardins emphasized the importance of comparing PCR probe data with chemical analysis. She observed “*PCR genotyping should be validated by chemical analysis of individuals that represent the allelic diversity of the target gene in the population*. *To avoid misinterpretation*, *it is critical to differentiate data obtained by genotyping from data obtained by chemical analysis*.” [21]. As noted above, the majority of studies on *F*. *graminearum* genotypes published today either determine the chemotype of a modest percentage of strains or no chemical analyses are reported at all. Thus the pattern of metabolites is only inferred, not determined.

**Fig 4:**
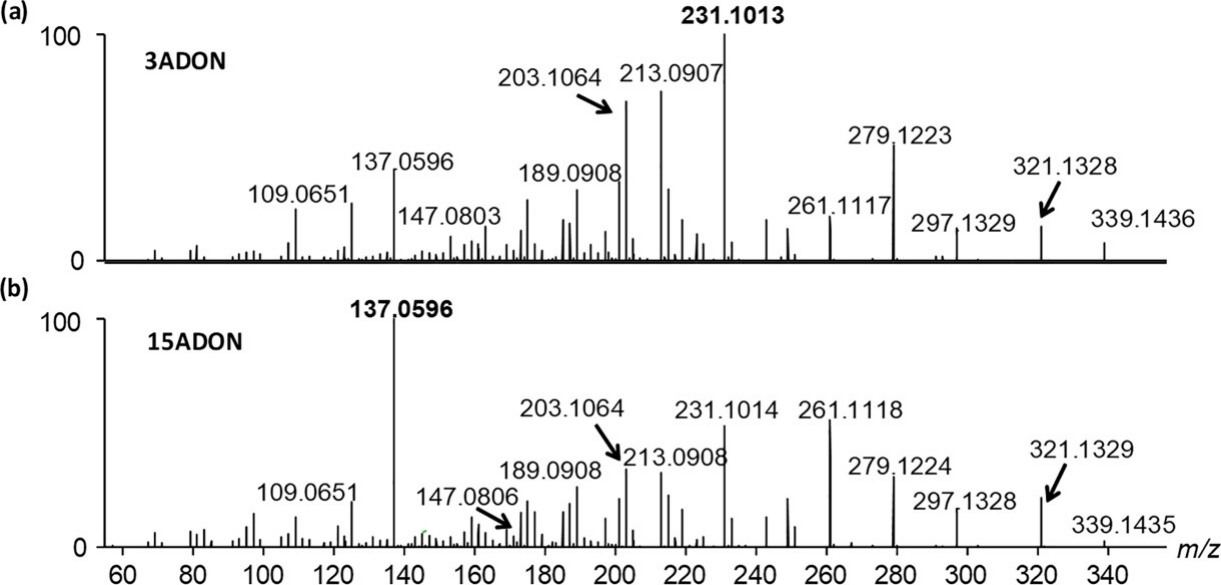
**MS/MS product ion spectra of protonated (a) 3ADON and (b) 15ADON.** All the major product ions produced by MS/MS of 3ADON are also produced by 15ADON at different proportions.

**Fig 5:**
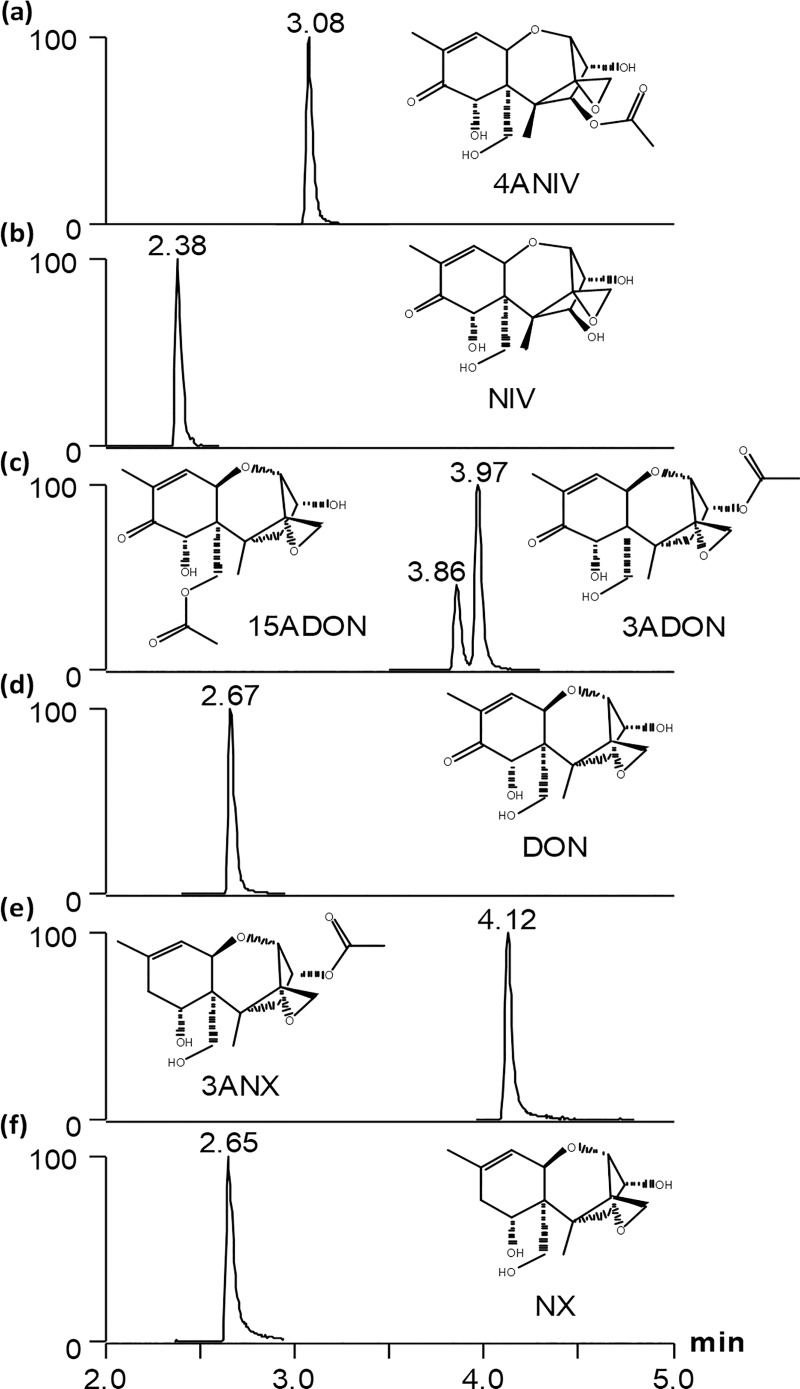
LC-MS/MS chromatograms of standard mix containing (a) 4ANIV, (b) NIV, (c) 15ADON and 3ADON, (d) DON, (e) 3ANX and (f) NX toxin monitored by settings in Table 2.

All of the trichothecenes listed in Table 2 can be distinguished by accurate mass alone, with exception of the position isomers 3ADON and 15ADON. A diagnostic feature in the MS/MS spectra of the two is that the *m/z* 137.0596 product ion has a much higher relative intensity in 15ADON than in 3ADON (Fig 4). For the monoacetylated DON precursors, the intensity ratio of *m/z* 137.1/231.1 < 1 for 3ADON and >1 for 15ADON. To further differentiate the two related compounds, we optimized the chromatographic conditions to resolve 15ADON and 3ADON (Fig 5C). This was accomplished using a 10 cm C18 column; narrow peak widths and resolution were achieved with a shallow gradient that began at 1 min with 22% organic phase ramping to 37% over 3 minutes. DON and NX toxin co-elute however, no in-source product ions of DON interfere with isolating the NX-H_2_O precursor ion were observed. This LC-MS/MS method also employed atmospheric pressure chemical ionization (APCI), which showed superior ionization of both the monoacetylated and non-acetylated target analytes.

**Table 2:** Optimized MS/MS settings for targeted analytes.

*compound*	*formula*	*ion type*	*RT (min)*	*precursor (m/z)*	*product ions : quantifier/qualifier (m/z)*	*collision energy (NCE)*
**3ADON**	C_17_H_22_O_7_	[M+H]^+^	3.97	339.1	231.1015/ 137.0597	28
**15ADON**	C_17_H_22_O_7_	[M+H]^+^	3.86	339.1	137.0597/ 231.1015	28
**DON**	C_15_H_20_O_6_	[M+H]^+^	2.69	297.1	203.1067/ 231.1017	29
**3ANX (NX-2)**	C_17_H_24_O_6_	[M+H]^+^	4.14	325.2	199.1116/ 137.0752	28
**NX (NX-3)**	C_15_H_22_O_5_	[M-H_2_O+H]^+^	2.67	265.1	105.0702/ 159.1167	38
**4ANIV**	C_17_H_22_O_8_	[M+H]^+^	3.10	355.1	229.0858/ 137.0597	26
**NIV**	C_15_H_20_O_7_	[M-H_2_O+H]^+^	2.40	295.1	137.0598/ 175.0754	37

The chemical screening method developed here allowed for highly confident detection of all trichothecenes needed to determine a strain’s chemotype. This facilitated the unexpected observation of 15ADON and 3ANX in the same culture extracts. The contention has been that production of NX represented strains with a unique genetic background producing only NX [42]. Broadly, most of the strains examined here had genotypes similar to the 15ADON control strain (DAOMC 180378) but were capable of producing both 15ADON and 3ANX. Each strain was determined to be a 15ADON genotype through *TRI3* and *TRI12* PCR assay. All but five (DAOMC 251912, 251914, 251915, 251919 and 251920) 15ADON and 3ANX producers were determined to be the 15ADON genotype with the *TRI8* assay. All but one (DAOMC 251918) were ADON genotype using the RFLP-PCR assay. There was no consistent pattern for 15ADON and 3ANX producers using the Toomajian assay. For this assay; 9/16 (56%) resulted in genotype matching the 15ADON producing control strain (DAOMC 180378), 4/16 (25%) resulted in genotype matching the 3ADON producing control strain (DAOMC 242075), 2/16 (13%) resulted in genotype matching the 3ANX producing control strain (DAOMC 242077). Overall, this confusing pattern suggests that these strains have Tri-cluster genotypes more similar to 15ADON strains than the known 3ANX strains.

This phenomenon has been observed by others. Sugiura et al. [56] reported a DON strain of *F*. *graminearum* resulting from a single ascospore that produced both DON and NIV. Kim et al. [57] reported some DON producing isolates from South Korea that also produced NIV. These authors could not determine differences in the three genes used to chemotype the strains between these and only DON producing isolates. They speculated that this might have resulted from recombination between two chemotypes [57]. Kulik et al. [58] reported 15ADON genotypes that produce large amounts of 3ADON as opposed to minor amounts from hydrolysis of 3,15ADON as the cultures become acidic due to the growth of the fungus. They reported that the strain had a Tri-cluster with an identical sequence to typical 15ADON chemotype strains. Earlier, Mirocha et al. [13] also reported a strain from Minnesota that produced material amounts of both acetates. The results of the present study and the work of others described above reflects the fact that as with other secondary metabolites, *Fusarium* trichothecenes are produced along metabolic grids [38]. Alternative pathways have arisen in nature and under experimental conditions through recombination during sexual reproduction [57], mutations, gains or losses in key biosynthetic genes such as *TRI1* [59], mutations that change precursor pools [37,38] or events unrelated to trichothecene production [60].

The available information at the time of the discovery of the 15ADON chemotype in 1983 through the subsequent decade was that it dominated the populations in the New World from Canada [8,11,61–63], through the temperate grain areas in the USA [12,13], Mexico [11] as well as South America [64,65]. In Canada, this picture has since evolved such that by 2008, the 3ADON population hitherto mainly found in Asia and Europe, became dominant in parts of western and eastern Canada [24]. The situation in the Canadian province of Ontario has been different; of *F*. *graminearum* strains isolated in a survey of Ontario, 97% of 155 isolates were the 15ADON genotype [66].

One possible explanation of the ability of some 15ADON producing strains to concomitantly produce 3ANX is a lowered specificity in two key enzymes, TRI1 and TRI8. The TRI1 enzyme of both 15ADON and 3ADON populations will produce 7,8-dihydroxycalonectrin (Fig 1). However, lowered specificity could also produce a portion of 7-hydroxycalonectrin, the precursor to 3ANX toxin. If a 15ADON strain produced some 7-hydroxycalonectrin, it might be anticipated that the specific activity of its TRI8 enzyme would produce 15-acetyl NX toxin (15ANX). Lacking chemical standards for 15ANX, it is not possible for us to conclude that these strains are not producing some amounts of 15ANX, however we can confidently state that they are producing 3ANX. For this to occur, the TRI8 enzyme of these strains would also need to have altered specificity.

The present data show that the capacity to produce 3ANX exists in and probably originated from the 15ADON background. In our opinion, this makes sense. As noted, the available data in the USA and Canada on the chemotype of *F*. *graminearum* strains through the mid 1990s produced were the15ADON chemotype meaning when wheat was analyzed for the two acetates of DON, 15ADON was present in the sample [12,62,67,68]. Studies of early collections of strains with the 15ADON background showed that these made a variety of calonectrin-related compounds [11,69].

From collections gathered in 1999 and 2000 from plant material showing FHB symptoms, Gale et al. [70] reported that the strains from US wheat areas were mainly of the 15ADON genotype with genetically divergent populations of strains in the upper Midwest USA. From samples collected between 1998–2001 in grain-growing areas of Western Canada, a genetically homogenous population of the 15ADON genotype was observed [71]. By the end of the 1990s, things were changing. The probes employed here were developed from strains mostly collected in the latter part of the 1990s in the USA. Using collections of strains made from 2005 and 2007 from Eastern to Western Canada, Kelly et al. [25] showed two large groupings of *F*. *graminearum* called NA1 (15ADON genotype) and NA2 (3ADON genotype). These authors reported that the frequency of recombinant genotypes is increasing rapidly. They further stated that trichothecene genotype is *“an increasingly unr*eli*able marker of population identity”*. Liang et al. [45] suggested that 3ANX strains fell within the NA2 or 3ADON population. Based on their work, Kelly et al. [25] suggested that that 3ANX strains may be a third genetic population but that *“much more work was needed to resolve this question”*.

Cropping over a large part of Ontario involves rotations of wheat, maize and soybeans and the region has experienced epidemic years for FHB or GER for over 100 years. These became more serious in the early 1980’s [72,73] and remains a chronic problem for either wheat or maize in Ontario. In the past 20 years, the ‘stay-green’ trait has become a common feature of maize hybrids with improved yields grown in Ontario [74]. When the crop is harvested, the stalks tend to be green offering an effective substrate for the production of *F*. *graminearum* ascocarps [75]. This perhaps explains the overall stability of the 15ADON genetic background yet accelerating recombination and the chemotype reported here, i.e. 15ADON/3ANX. This is consistent with two recent studies of the genetic diversity in *F*. *graminearum* populations. Kelly & Ward, [76] reported that full genome scans found frequently recombining regions notably with high genomic divergence of the trichothecene toxin gene cluster; Yue reported a similar phenomenon [52].

The present data demonstrate that *F*. *graminearum* isolates that are able to produce both 15ADON and 3ANX are common in Ontario. This is a region where the 3ADON strains have never been common. The 15ADON strain used here, DAOMC 180378 did not produce detectable levels of 3ANX. The present data do not shed light on whether the 15ADON/3ANX chemotype was present 40 years ago. Although a pathway has been inferred for the accumulation of 3ANX, experience with other *Fusarium* species is clear that more than one pathway can exist within a population. More than 80% of our strains produced some 3ANX in addition to 15ADON. Comparison of chemical data with the methods used for genotyping revealed that in more than 50% of the cases there was no clear agreement. The seemingly rapid change in *F*. *graminearum* populations evidenced by both these chemical data and the genetic data of Kelly & Ward, [76] explain why the common PCR probes for genotype only work sometimes. With appreciation to Varga et al. [42] this old enemy really does have new tricks. The data presented here, demonstrate that the available genomic tools do not provide the complete picture as to the toxicogenic potential of *F*. *graminearum* strains. As such we are arguing that chemical analysis is critical for all future studies of this fungus and the use of LC-MS/MS methods such as the one described here that are capable of differentiating all of the known compounds are necessary. Strain data from all three years of the study will be examined for any influence of agronomy on chemotypes recovered in a subsequent report.

## Supporting information

S1 Table Location and GPS coordinates of representative strains collected from Ontario, Canada in 2015. (PDF)
