# Supplementary material for: Comparing genotype and chemotype of Fusarium graminearum from cereals in Ontario, Canada
Source: PLoS One. 2019 May 9;14(5):e0216735. doi: 10.1371/journal.pone.0216735 (PMC6508712; doi:10.1371/journal.pone.0216735)
Supplement: S1 Table — (PDF) [file pone.0216735.s001.pdf]

Supporting information

S1 Table. Location and GPS coordinates of representative strains collected from Ontario, Canada in 2015.

DAOMC	Accession number	Location	Latitude	Longitude
251903	MH108123	Sarnia, Lambton	42.94	-82.25
251904	MH108124	Essex, Essex	42.13	-82.91
251905	MH108125	Palmerston, Wellington	43.87	-80.83
251906	MH108126	Belle River, Essex	42.29	-82.64
251907	MH108127	Denfield, Middlesex	43.14	-81.26
251908	MH108128	Inwood, Lambton	42.81	-81.91
251909	MH108129	Muirkirk, Chatham-Kent	42.53	-81.74
251910	MH108130	Duart, Chatham-Kent	42.50	-81.76
251911	MH108131	Ridgetown, Chatham-Kent	42.44	-81.88
251912	MH108132	Blenheim, Chatham-Kent	42.36	-82.07
251913	MH108133	Melbourne, Middlesex	42.86	-81.55
251914	MH108134	Dresden, Chatham-Kent	42.58	-82.13
251915	MH108135	Lakeshore, Essex	42.30	-82.64
251916	MH108136	Exeter, Huron	43.13	-81.24
251917	MH108137	Huron East, Huron	43.47	-81.45
251918	MH108138	Dublin, Perth	43.47	-81.25
251919	MH108139	Mitchell, Perth	43.13	-81.24
251920	MH108140	Lucan, Middlesex	43.23	-81.40
251921	MH108141	Florence, Lambton	42.71	-81.96
251922	MH108142	Rodney, Elgin	42.55	-81.62
